# Cholesterol induces inflammation and reduces glucose utilization

**DOI:** 10.1515/med-2023-0701

**Published:** 2023-05-12

**Authors:** Pingping Hong, Qing Wang, Guoping Chen

**Affiliations:** Department of Endocrinology, Shaoxing Central Hospital, Shaoxing 312000, Zhejiang, P.R. China; Department of Clinical Laboratory Centre, Shaoxing People’s Hospital, Shaoxing 312000, Zhejiang, P.R. China; Department of Endocrinology, Deqing People’s Hospital, No. 120 Yingxi South Road, Wukang Town, Deqing County, Huzhou City 313200, Zhejiang, P.R. China

**Keywords:** diabetes mellitus, pancreatic, cholesterol, inflammatory, endoplasmic reticulum stress

## Abstract

Cholesterol stimulates inflammation and affects the normal function of islet tissues. However, the precise mechanism underlying the effects of cholesterol on islet cells requires clarification. In this study, we explored the role of cholesterol in glucose utilization in pancreatic cells. Beta-TC-6 cells and mice were treated with cholesterol. We used glucose detection kits to identify the glucose content in the cell culture supernatant and mouse serum and an enzyme-linked immunosorbent assay was used to detect insulin levels in the serum. Glucose-6-phosphatase catalytic subunit 2 (G6PC2), 78 kDa glucose-regulated protein (GRP78), 94 kDa glucose-regulated protein (GRP94), nucleotide-binding oligomerization domain-like receptor protein 3 (NLRP3), caspase-1 (casp1), and interleukin-1β (IL-1β) expression levels were detected using immunofluorescence, immunohistochemistry, western blotting, and reverse transcription-quantitative polymerase chain reaction. Hematoxylin–eosin staining was used to detect the histological alterations in pancreatic tissues. Cholesterol decreased beta-TC-6 cell glucose utilization; enhanced pancreatic tissue pathological alterations; increased glucose and insulin levels in mouse serum; increased G6PC2, GRP78, GRP94, and NLRP3 expression levels; and elevated casp1 and pro-IL-1β cleavage. Cholesterol can attenuate glucose utilization efficiency in beta-TC-6 cells and mice, which may be related to endoplasmic reticulum stress and inflammation.

## 1 Introduction

Diabetes mellitus (DM), a common worldwide disease, is a metabolic disorder characterized by high blood sugar [1]. A prevalence survey estimated that there were 463 million DM patients in 2019 globally and predicted that this number would rise to 700 million over the next 25 years [2]. Type 1, type 2, and gestational DM are the three primary categories of diabetes [3]. Type 2 DM results from a gradual loss of insulin sensitivity and production, whereas type 1 DM is caused by an autoimmune illness that attacks the patient’s insulin-producing β-cells [4]. Only pregnant women can be diagnosed with gestational diabetes, which has a similar etiology to type 2 diabetes [3]. Despite differences in the etiologies of type 1 and 2 diabetes, pancreatic β-cell dysfunction and apoptosis are essential for both forms to develop [5,6,7].

Studies have demonstrated that cellular lipids and cholesterol availability impact several processes that control insulin synthesis, storage, and secretion [8]. Cell formation and differentiation depend on cholesterol, a component of cell membranes and a precursor of bile acids, steroid hormones, and vitamin D [9,10]. Numerous findings suggest that cellular cholesterol buildup may cause pancreatic β-cell failure. Patients with type 2 diabetes have a collection of lipid abnormalities linked to fatty acid and cholesterol buildup in pancreatic β-cells that may impact the progression of pancreatic islet degeneration [11,12] and harm the insulin secretion mechanism [13]. Therefore, cholesterol levels inside β-cells must remain below predetermined ranges to ensure insulin secretion. Cholesterol is synthesized in the endoplasmic reticulum. *De novo* cholesterol synthesis in the endoplasmic reticulum and uptake of cholesterol-containing low-density lipoproteins (LDLs) constitute a well-coordinated mechanism of cholesterol homeostasis [14]. Existing studies have clarified that endoplasmic reticulum stress (ERS) is one important reason for islet β-cell dysfunction and apoptosis [15]. An increase in free cholesterol inside the cells leads to ERS [13]. Moderate ERS is a self-protection mechanism for cells to respond to environmental stimuli, whereas persistent ERS may aggravate inflammatory responses and even lead to premature cell apoptosis and necrosis [16,17].

Recently, the role of inflammation in the pathophysiology of diabetes has received considerable attention. Inflammation is believed to be an important part of the pathogenesis of diabetes, which is considered a chronic low-grade inflammatory disease [18]. Insulin resistance caused by inflammatory responses increases the risk of type 2 diabetes, which further exacerbates hyperglycemia, leading to long-term diabetes consequences [19,20]. Various inflammatory factors and proteins, such as interleukins (ILs), C-reactive protein, and tumor necrosis factor-α, can intensify insulin resistance by inhibiting insulin receptor tyrosine kinase activity and participate in diabetes development [21]. The inflammasome, which is mostly composed of nucleotide-binding oligomerization domain-like receptor protein 3 (NLRP3), is a crucial regulator that controls immunological inflammation and cell death in the body. The apoptosis-associated speck-like protein (ASC) with a recruitment domain and effector proteins caspase-1 (casp1), IL-1β, and IL-18 is vital in various inflammatory immune diseases, including type 2 diabetes, through cascading activation of the NLRP3-ASC-casp1-IL-1β/IL-18 axis upon corresponding stimulation [22,23]. Additionally, cholesterol is the main stimulating factor activating the NLRP3 inflammasome. LDL cholesterol (LDL-C) can activate the NLRP3 inflammasome in macrophages, encouraging an inflammatory response [24]. Both high hyperglycemia and lipopolysaccharide levels may activate the NLRP3 inflammasome in mesangial cells through the reactive oxygen species (ROS)/thioredoxin-interacting protein (TXNIP) pathway [18]. Concurrently, cholesterol is the main target of NLRP3 inflammasome activation. Interfering with NLRP3 gene expression significantly delays atherosclerosis progression in apolipoprotein E-deficient mice and inhibits plaque formation on the vessel wall [25].

However, the specific regulatory mechanisms of inflammation and cholesterol remain unclear, and the specific mechanism of cholesterol in islet cells requires elucidation. Based on our research, we constructed a model of cholesterol in islet cells to observe the relevance of cholesterol in inflammation and ERS in pancreatic tissue cells *in vivo* and *in vitro* and explore the influence of glucose and insulin release.

## 2 Methods

### 2.1 Cell culture and treatment

Beta-TC-6 cells were purchased from IMMOCELL (catalog number: IM-M053, Xiamen, China) and treated with Dulbecco’s modified Eagle’s medium containing 15% fetal bovine serum at 37°C in a 5% CO_2_ environment. When the cell confluence reached approximately 80%, the cells were divided into two groups according to the intervention technique: the mock group, which received treatments without cholesterol, and the cholesterol group, which underwent a 6 h pretreatment with 5 mM cholesterol (catalog number: C4951; Sigma-Aldrich).

### 2.2 Immunofluorescence

The cells were fixed with 4% formaldehyde (catalog number: 10010018; Sinopharm Chemical Reagent Co., Ltd.) for 30 min and sealed with 5% bovine serum albumin (BSA) (catalog number: A8010; Solarbio). The treated cells were then incubated with 0.5% Triton X-100 (catalog number: T8210; Solarbio) for 20 min at 25°C. The cells were stained with anti-glucose-6-phosphatase catalytic subunit 2 (G6PC2) antibody (catalog number: CSB-PA873624LA01HU, 1:100; CUSABIO) overnight at 4°C and stained an hour later at 37°C in the dark with Alexa Fluor® 488-labeled goat anti-rabbit IgG (catalog number: GB25303, 1:100; Servicebio). 4′,6-Diamidino-2-phenylindole (DAPI) (catalog number: S2110; Solarbio) was used to stain the nuclei for 10 min. Images were taken using a fluorescence microscope (model number: DMIL LED; Leica).

### 2.3 Experimental animals

We purchased 12 7–8-week-old male C57BL/6 mice (each weighing between 18 and 20 g) from the Laboratory Animal Center of China Three Gorges University. The animals had access to water and a regular meal while being grown in a comfortable habitat at a temperature of 23°C in a natural 12 h/12 h light/dark cycle (lights on at 08:00; lights off at 20:00). Ethical approval was granted by the Animal Care and Use Committee of Zhejiang University.

### 2.4 Mice experimental design

After an 8 h fast, six C57BL/6 mice were randomly selected (mock group) and given regular food and distilled water to drink to represent the blank control group. The remaining six C57BL/6 mice were assigned to the cholesterol group, which received distilled water, a regular diet, and cholesterol at a dose of 10 mg/(kg/day) via gavage. After 4 weeks, all the mice were sacrificed using sodium pentobarbital (150 mg/kg). Blood and pancreatic tissues were collected.

### 2.5 Reverse transcription-quantitative polymerase chain reaction (RT-qPCR)

Total RNA was extracted from beta-TC-6 cells and mouse pancreatic tissues using the TRIzol® reagent (catalog number: 15596-026, Ambion), and cDNA was produced using a cDNA synthesis kit (catalog number: R223-01, VAZYME) as per the manufacturer’s instructions. qPCR was performed using SYBR Green Master Mix (catalog number: Q111-02; VAZYME). Table 1 lists the primers for RT-qPCR. The relative expression levels of the target genes were calculated using the 2^−ΔΔCq^ method.

**Table 1:** Primer sequence list of RT-qPCR

Gene	Primer	Sequence (5′–3′)	PCR products
Mus GAPDH	Forward	ATGGGTGTGAACCACGAGA	229 bp
	Reverse	CAGGGATGATGTTCTGGGCA	
Mus NLRP3	Forward	TCTCCCGCATCTCCATTTGT	223 bp
	Reverse	CTGTCCCGCATTTTAGTCCG	
Mus Caspase1	Forward	CAGGAGGGAATATGTGGG	120 bp
	Reverse	CACCTTGGGCTTGTCTTT	
Mus IL-1β	Forward	TCAGGCAGGCAGTATCACTC	250 bp
	Reverse	AGCTCATATGGGTCCGACAG	
Mus G6PC2	Forward	ACATTGACAGCACGCCTTTT	248 bp
	Reverse	GGGGATGGACGCACTTTTAC	
Mus GRP78	Forward	ATTGTTCTGGTTGGTGGA	348 bp
	Reverse	TTTTGTTAGGGGTCGTTC	
Mus GRP94	Forward	TGTGTGGGATTGGGAACT	354 bp
	Reverse	GAAACATTGAGGGGGAGA	

### 2.6 Western blotting

Tissues or cells were lysed with radioimmunoprecipitation assay lysate (catalog number: P0013B; Beyotime) to extract proteins whose concentrations were measured using the bicinchoninic acid assay method (catalog number: P0010; Beyotime). The protein samples (50 μg) underwent sodium dodecyl sulfate polyacrylamide gel electrophoresis; they were then transferred to polyvinylidene difluoride membranes (catalog number: IPVH00010; Millipore) and incubated with 50 mg/mL skimmed milk powder for 1 h. They were incubated with the primary antibody at room temperature for 1 h or overnight at 4°C. After incubating with the secondary antibody at room temperature for 1–2 h, enhanced chemiluminescence substrate solution (catalog number: P1050; APPLYGEN) was added dropwise to make the band appear. Grayscale analysis and statistics were performed using ImageJ software. Table 2 lists the antibody information.

**Table 2:** Antibody information for western blotting and immunohistochemistry

Classification	Antibodies	Manufacturer	Catalog number
Primary antibodies	Anti-GAPDH	Goodhere	AB-P-R001
	Anti-G6PC2	CUSABIO	CSB-PA873624LA01HU
	Anti-GRP78	Proteintech	66574-1-Ig
	Anti-GRP94	Proteintech	14700-1-AP
	Anti-NLRP3	Affinity Biosciences	DF7438
	Anti-Caspase-1	AdipoGen	AG-20B-0042-C100
	Anti-IL-1β	Affinity Biosciences	AF5103
Second antibody	HRP-conjugated Goat anti-rabbit IgG	Proteintech	SA00001-2
	HRP-conjugated goat anti-mouse IgG	Proteintech	SA00001-1

### 2.7 Insulin detection

According to the reagent operation instructions, a mouse insulin enzyme-linked immunosorbent assay (ELISA) kit (catalog number: E-EL-M1382c; Elabscience Biotechnology Co., Ltd.) was used to detect the amount of insulin in the serum. Absorbance was measured at 450 nm using a multifunctional enzyme marker (model number: Flexstation3; Molecular Devices).

### 2.8 Glucose detection

The glucose concentration in the cell culture supernatant and serum was determined using a glucose determination kit (catalog number: F006-1-1; Shanghai Rongsheng Bio-Pharmaceutical Co., Ltd.) according to the manufacturer’s instructions. A multifunctional enzyme marker (model number: Flexstation3; Molecular Devices) was used to measure the absorbance at 505 nm.

### 2.9 Hematoxylin–eosin staining

After fixation in a 4% formaldehyde solution and dehydration in ethanol, the pancreatic tissues were embedded in paraffin and cut into a 4 μm section. After deparaffinization, the sections were stained with a hematoxylin–eosin staining kit (catalog number: G1076, Servicebio) and observed under a light microscope (Nikon).

### 2.10 Immunohistochemistry

The paraffin sections of fixed pancreatic tissues were created, and then roasted, deparaffinized, and rehydrated before being steamed for 30 min in a 0.01 mol/L citric acid/sodium citrate buffer to perform antigen retrieval. The sections were incubated in 3% hydrogen peroxide at room temperature for 15 min to remove endogenous peroxidase activity and then incubated in 5–10% goat serum at 37°C for 15 min. After incubation with primary antibodies overnight at 4°C, the section was incubated with the horse radish peroxidase-labeled secondary antibody at room temperature for 30 min and then stained with the DAB and hematoxylin at room temperature for 1–3 min. A light microscope was used to capture the images. Table 2 shows all the antibody information.

### 2.11 Statistical analysis

D’Agostino & Pearson test and Shapiro–Wilk tests were used to assess the normality of data. The F test was used to assess the homogeneity of variances. Two groups were compared using an unpaired Student’s *t*-test with GraphPad Prism software (version 8.0). Statistical significance was set at *P* < 0.05.

## 3 Results

### 3.1 Cholesterol decreases glucose utilization and enhances G6PC2 expression in beta-TC-6 cells

**Figure 1:**
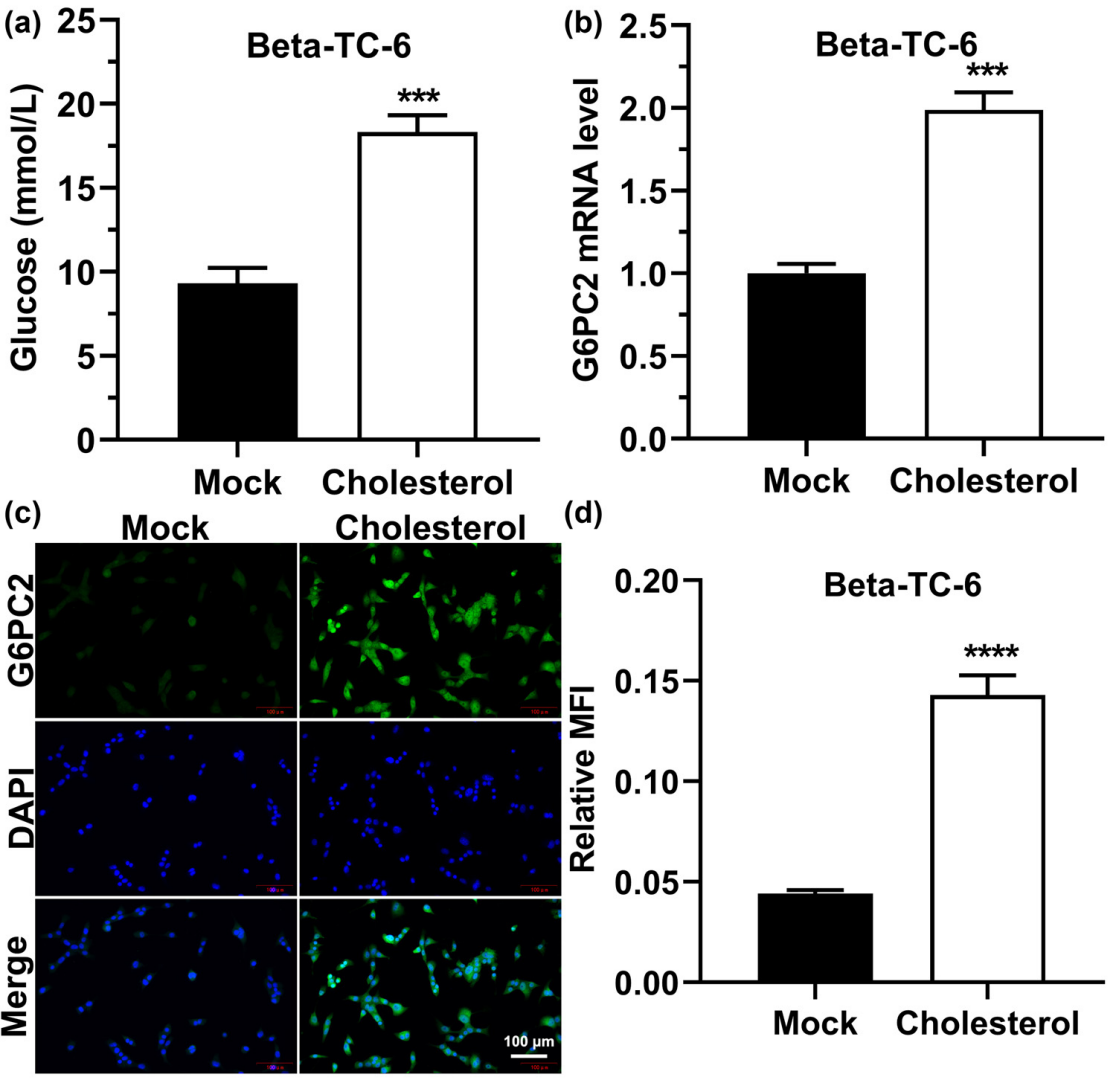
Effect of cholesterol on glucose utilization by beta-TC-6 cells. (a) After beta-TC-6 cells were treated with cholesterol, the glucose content in the cell culture supernatant was detected. (b) After the beta-TC-6 cells were treated with cholesterol, the G6PC2 mRNA level was detected using RT-qPCR. (c) After the beta-TC-6 cells were treated with cholesterol, the G6PC2 protein level was detected using an immunofluorescence experiment. (d) Statistical analysis of green fluorescence in (c). “MOCK” indicates the control group, *P < 0.05, **P < 0.01, ***P < 0.001, ****P < 0.0001.

We treated beta-TC-6 cells with 5 mM cholesterol to detect the effect of cholesterol on the pancreatic cell function and found that the glucose content in the supernatant of the cells was significantly higher than that in the supernatant of cells treated without cholesterol (Figure 1a). G6PC2 encodes a member of the catalytic subunit family of glucose-6 phosphatases, which hydrolyzes glucose-6-phosphate to glucose, possibly by participating in glucose production through gluconeogenesis and glycogen decomposition [26,27]. Therefore, G6PC2 genes and proteins were examined using RT-qPCR and immunofluorescence. We found that the cholesterol treatment significantly increased G6PC2 expression levels (Figure 1b–d). These results indicated that cholesterol treatment increased the expression of G6PC2 and decreased the glucose utilization rate in beta-TC-6 cells.

### 3.2 Cholesterol increases the expression of inflammatory- and ERS-related molecules in beta-TC-6 cells

**Figure 2:**
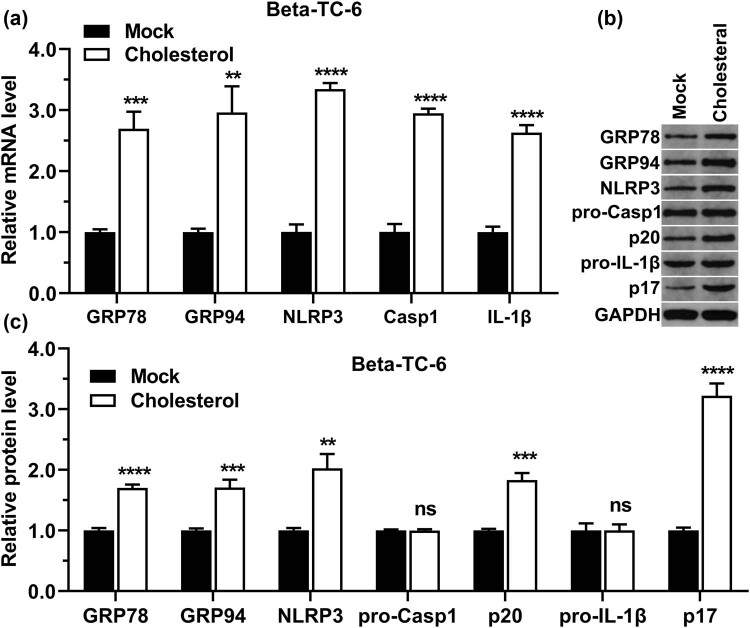
Effects of cholesterol on the inflammatory response and the expression of endoplasmic reticulum-related molecules in beta-TC-6 cells. (a) After beta-TC-6 cells were treated with cholesterol, GRP78, GRP94, NLRP3, casp1, and IL-1β mRNA levels were detected using RT-qPCR. (b) After beta-TC-6 cells were treated with cholesterol, GRP78, GRP94, NLRP3, pro-casp1, pro-IL-1β, p20 (cleaved casp1), and p17 (cleaved IL-1β) protein levels were analyzed by western blot, using GAPDH as an internal reference. (c) Statistical analysis of the western blot experiments in (b). “MOCK” indicates the control group, *P < 0.05, **P < 0.01, ***P < 0.001, ****P < 0.0001.

We assessed the expression of inflammatory- and endoplasmic reticulum-related genes, including NLRP3, casp1, IL-1β, GRP78, and GRP94, in cholesterol-treated beta-TC-6 cells using RT-qPCR. The results showed that cholesterol increased GRP78, GRP94, NLRP3, casp1, and IL-1β mRNA levels in beta-TC-6 cells (Figure 2a). Western blotting results revealed that the protein levels of GRP78, GRP94, NLRP3, p20 (cleaved casp1), and p17 (cleaved IL-1β) were boosted in cholesterol-treated beta-TC-6 cells (Figure 2b and c). The above results show that cholesterol activated the inflammatory response in beta-TC-6 cells.

### 3.3 Cholesterol damages the islet structure of the mice, increases blood glucose and insulin levels, and enhances the expression of G6PC2 in the pancreas

**Figure 3:**
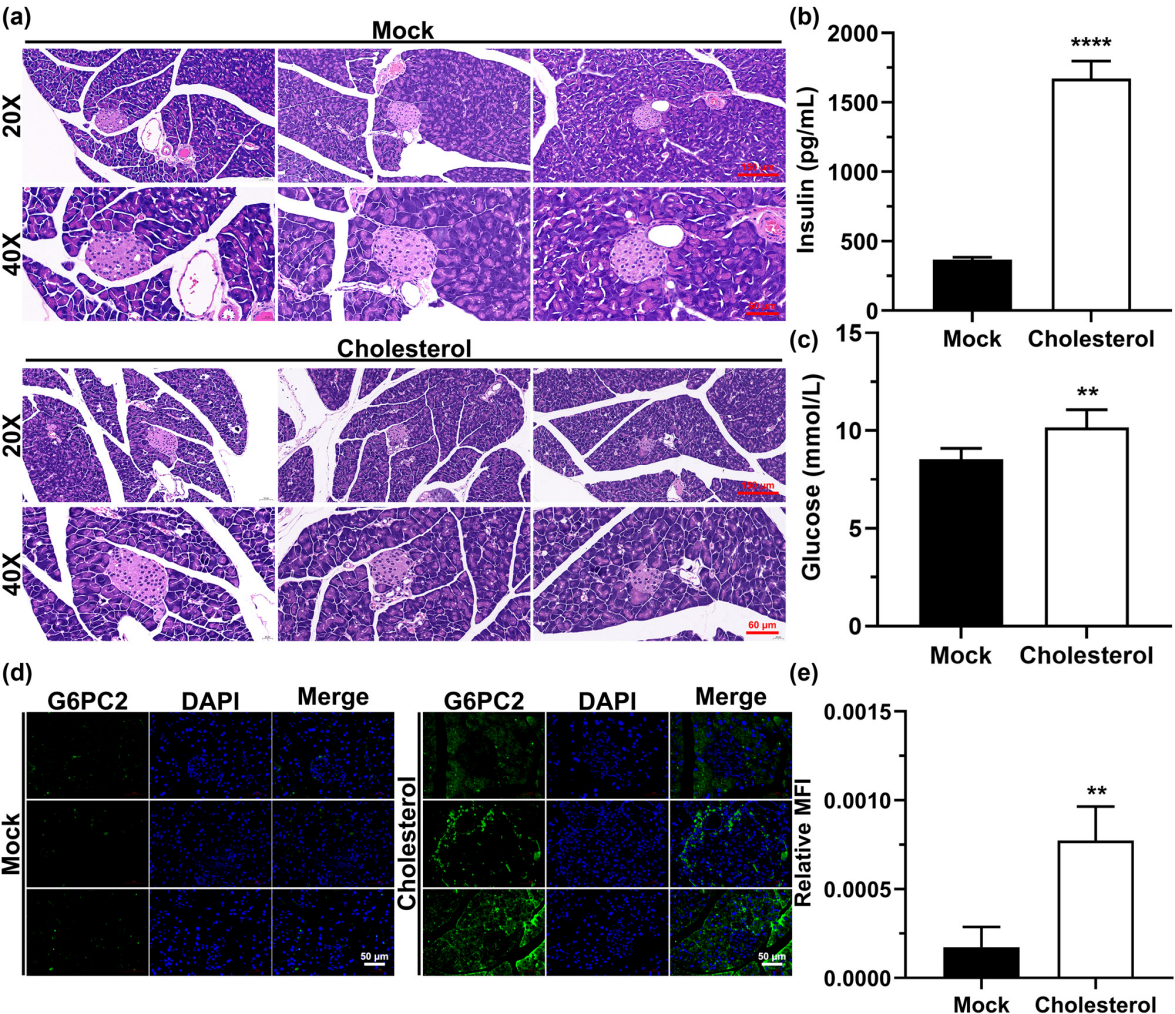
Effects of cholesterol on mice pancreas. (a) After the mice were treated with cholesterol, the lesions of the pancreatic tissue were observed using hematoxylin–eosin staining. (b) After the mice were treated with cholesterol, the insulin content in their blood was detected by ELISA. (c) After the mice were treated with cholesterol, the glucose content in their blood was detected. (d) Immunofluorescence was used to observe the expression of the G6PC2 protein in mice pancreas after they were treated with cholesterol. (e) Statistical analysis of green fluorescence in (d). “MOCK” indicates the control group, *P < 0.05, **P < 0.01, ***P < 0.001, ****P < 0.0001.

We constructed a cholesterol mouse model to investigate the role of cholesterol in pancreatic tissues. According to the hematoxylin–eosin staining results, the islet tissues of the cholesterol-free group had clean boundaries, a full structure, and no necrosis, hemorrhage, or inflammatory cell infiltration, and were round or oval in shape (Figure 3a). In contrast, the pancreatic islet tissues of the cholesterol-treated group showed an incomplete structure, disordered pancreatic islet cell arrangement, and a cord-like or irregular shape (Figure 3a). In addition, the levels of insulin and glucose in the blood of the mice, and the G6PC2 protein expression in the mouse pancreas were significantly higher than that of the control group (Figure 3b–e). These results indicate that cholesterol damaged the mouse pancreas and increased blood glucose concentration.

### 3.4 Cholesterol increases the expression of inflammatory- and ERS-related molecules in mice

**Figure 4:**
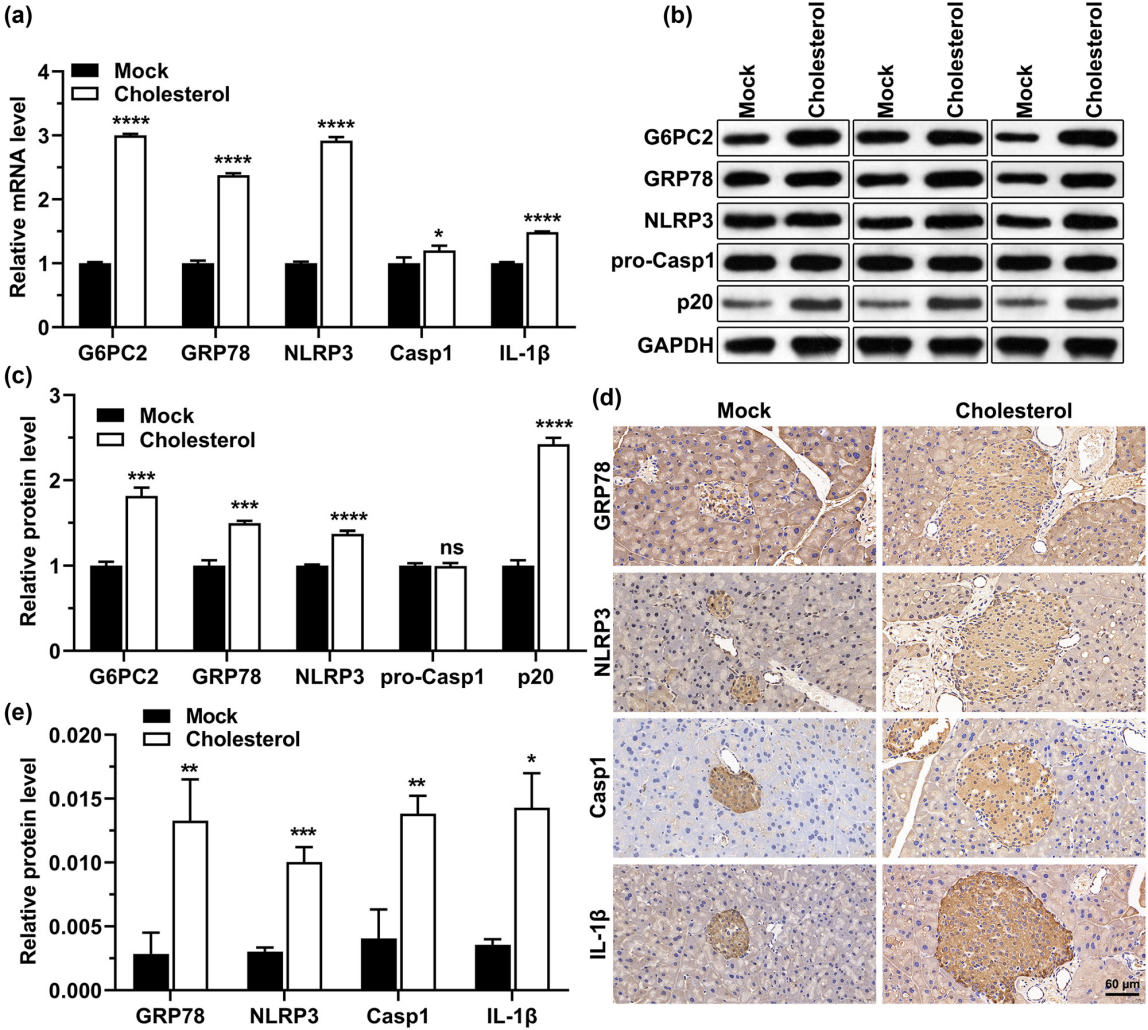
Effects of cholesterol on the inflammatory response and the expression of endoplasmic reticulum-related molecules in mice pancreas. (a) After the mice were treated with cholesterol, G6PC2, GRP78, NLRP3, casp1, and IL-1β mRNA levels were detected using RT-qPCR. (b) After the mice were treated with cholesterol, GRP78, G6PC2, NLRP3, pro-casp1, and p20 (cleaved casp1) protein levels were detected by western blot, using GAPDH as an internal reference. (c) Statistical analysis of the western blot experiments in (b). (d) After the mice were treated with cholesterol, GRP78, NLRP3, casp1, and IL-1β protein levels were detected via immunohistochemistry. (e) Statistical graph of immunohistochemistry analysis in (d). “MOCK” indicates the control group, *P < 0.05, **P < 0.01, ***P < 0.001, ****P < 0.0001.

We also detected the expression of inflammatory- and endoplasmic reticulum-related molecules in the mice pancreas using RT-qPCR. The results showed that cholesterol significantly increased G6PC2, GRP78, NLRP3, casp1, and IL-1β mRNA and protein levels in the mice pancreas (Figure 4a–d). These findings imply that cholesterol may contribute to inflammation and ERS.

## 4 Discussion

Numerous medications and surgical procedures are used to treat diabetes but their outcomes have been unsatisfactory. Several researchers are striving to dive further into the pathologic foundations of the disease to discover more effective treatment options [5,7,8,13]. Cholesterol is a crucial component of cell membranes that helps regulate their physical characteristics. It is also essential for the proper operation of pancreatic β-cells. According to growing experimental data, the components affecting cellular cholesterol metabolism are thought to affect the β-cell function and the pathogenesis of diabetes [8,9,10,14]. Additionally, recent research has shown that cholesterol metabolism and β-cell activity are intimately connected, and the balance of cholesterol metabolism is necessary for β-cell functions, including insulin production, and apoptosis may occur if this balance is upset [28]. In this study, we found that exogenous excess cholesterol induced ERS in beta cells and activated inflammatory responses, thereby damaging pancreatic functions.

ERS is a well-known factor in the development of diabetes [16] that can be induced by cholesterol to some extent [29]. Pancreatic β-cells have highly developed ERS and a strong endoplasmic reticulum signaling system because of their high levels of specialization in insulin production and secretion. As a result, they are particularly vulnerable to ERS when the amount of insulin produced exceeds the endoplasmic reticulum’s capacity to fold proteins. In contrast, one of the most crucial functions of β-cells is insulin secretion, which is reduced by cholesterol exposure [28,30]. However, this study found that after cholesterol stimulation, serum insulin secretion in mice increased and potentially aggravated ERS, resulting in the abnormal expression of glucose-regulated proteins (GRPs), such as GRP78 and GRP94. Another theory is that G6PC2 dephosphorylates glucose-6-phosphate back to glucose, thereby creating a glucose cycle. We found that G6PC2 expression was considerably upregulated following cholesterol treatment. Insulin secretion pulsatility may be disrupted by G6PC2 variations, which would decrease the effectiveness of insulin transmission between the pancreas and the liver. There may be a slight increase in absolute insulin secretion due to the slight increase in glucose caused by hepatic insulin resistance [31].

In addition, elevated blood glucose levels trigger the metabolic activity of islet cells, leading to the production of more ROS by the mitochondria [18]. Generating ROS promotes NLRP3 inflammasome and capsidase-1 activation, which are also associated with the NLRP3 pathway, leading to increased IL-1β release. The present study observed a similar phenomenon after the cholesterol treatment. In addition, lipopolysaccharides bound to fetuin-A in the blood also increase toll-like receptor (TLR)2 and TLR4 activation, leading to the translocation of nuclear factor-κB (NF-κB), TXNIP, and the production of pro-inflammatory cytokines. Additionally, activated casp1 cleaves IL-1β and IL-18 precursors to form active IL-1β and IL-18, which are released extracellularly, recruits inflammatory cells to aggregate, and expands the inflammatory response [7]. Similarly, in this experiment, the cholesterol significantly increased the protein levels of casp1, pro-IL-1β, cleaved casp1, and cleaved IL-1β, and may have eventually participated in pyroptosis.

In summary, in beta-TC-6 cells and mice, cholesterol can attenuate glucose utilization efficiency, increase glucose and insulin levels, and enhance the expression of G6PC2. Moreover, cholesterol damages the islet structure of the mice. These data suggest that excessive cholesterol intake can trigger inflammatory responses and ER stress, leading to pancreatic damage and reduced glucose utilization, which provides a novel insight into the pathogenesis of diabetes.
